# Green synthesis and antioxidant evaluation of calcium oxide nanoparticles using *Syzygium cumini* fruit extract: An *in vitro* study

**DOI:** 10.6026/973206300223338

**Published:** 2026-06-30

**Authors:** Srividhya Srinivasan, K Saraswathi Gopal, Shanmugam Rajesh Kumar, Anand B, Thirumal Kumar

**Affiliations:** 1 Department of Oral Medicine and Radiology, Faculty of Dentistry, Meenakshi Ammal Dental College and Hospital, Meenakshi Academy of Higher Education and Research, Chennai, Tamil Nadu, India; 2 Nano biomedicine Lab, Centre for Global Health Research, Saveetha Institute of Medical and Technical Sciences, Chennai, Tamil Nadu, India; 3 Department of Oral Medicine and Radiology, Ragas Dental College and Hospital, Chennai, Tamil Nadu, India; 4 Central Research laboratory, Meenakshi Academy of Higher Education and Research, Chennai, Tamil Nadu, India

**Keywords:** *Syzygium cumini*, green *synthesis calcium* oxide nanoparticles, nanobiotechnology, antioxidant activity, free radical scavenging

## Abstract

The lack of safe, cost-effective, and eco-friendly antioxidant agents to combat oxidative stress-induced chronic diseases remains a significant challenge in biomedical research. Green synthesis offers a sustainable approach for producing bioactive nanoparticles using plant-derived phytochemicals. In this study, calcium oxide nanoparticles (CaO NPs) were synthesized using *Syzygium cumin* fruit extract. The formation of nanoparticles was confirmed by UV-Visible spectroscopy, while X-ray diffraction (XRD), transmission electron microscopy (TEM), and Fourier transform infrared spectroscopy (FTIR) revealed crystalline, spherical nanoparticles stabilized by phytochemical capping. The antioxidant activity of the synthesized CaO NPs was evaluated using DPPH, FRAP, ABTS, hydrogen peroxide, and nitric oxide assays at concentrations ranging from 10-50 μg/mL. The nanoparticles exhibited strong, concentration-dependent radical scavenging activity comparable to ascorbic acid. Data shows the potential of S. cumin-mediated CaO NPs as promising candidates for biomedical applications targeting oxidative stress. Thus, we show that *Syzygium cumini*-mediated green synthesis can produce structurally stable CaO nanoparticles with significant antioxidant activity, thereby contributing to the development of eco-friendly nanomaterials for oxidative stress-related biomedical applications

## Background:

Green synthesis, the environmentally friendly synthesis or sustainable synthesis, refers to the development of processes and methodologies that minimizes or avoids the use of harmful substances and reduce the production of waste [1]. It has achieved significant attention in pharmaceutical and biomedical research, as researchers are looking for green methods for the synthesis of bioactive compounds and novel therapeutic agents. *Syzygium cumini* is also known as jamun or Indian blackberry. It is a system of traditional medicine which utilizes different parts of the plant in producing medicines found to be beneficial for treating diabetes mellitus, hyperglycemia, and hyperlipidemia. Numerous other parts of the plant including fruit and seeds have certificated pharmacological applications, such as antidiabetic, anticancer, antioxidant and antimicrobial properties [2, 3]. Due to its abundant phytochemicals including polyphenols, flavonoids, anthocyanins and tannins; the fruit extract has been exploited as a potential green reducing and stabilizing agent for nanoparticle synthesis. Nanotechnology has become a revolutionizing platform in current therapeutics, with respect to regenerative medicine, drug delivery and wound management [4]. Nanoparticles possess distinct advantages owing to their nanoscale size and high surface-area-to-volume ratio, increasing their reactivity, bioavailability, and ability to transport drugs across biological barriers [5]. Calcium oxide (CaO) nanoparticles are known for their biocompatibility, physiological stability, and suitable application for biomedical applications. Their modifiable physicochemical characteristics including particle size, surface charge, and morphology increases improved drug loading efficiencies and controlled release function. Known for phytochemical richness of *S. cumini* and the functional qualities of CaO nanoparticles, integrating green synthesis with nanotechnology presents a promising method for developing biologically active and safe nanomaterials. Therefore, it is of interest to explore *S. cumini* fruit extract, a natural reducing and capping agent to synthesize calcium oxide nanoparticles which was subsequently evaluated for its antioxidant property to determine its potential applicability in therapeutic and biomedical settings.

## Methodology:

*In vitro* study, Chennai, conducted between June to September 2025, fresh, ripe *Syzygium cumini* fruits were washed thoroughly, and the seeds were removed. Approximately 5 g of the deseeded fruit pulp was weighed and manually ground using a mortar and pestle to obtain a uniform, semi-solid paste. The crushed pulp was then filtered through a muslin cloth to remove fibrous material, and the resulting filtrate was added to the distilled water to reach up to 50 mL and aqueous fruit extract was obtained. The fruit extract was boiled for 10 minutes at 30 °C. The boiled extract was used to prepare the nanoparticles. Preparation of *S. cumini* - CaO NPs 100mM of calcium hydroxide solution was prepared for 50mL. 50mL of fruit extract was mixed with calcium hydroxide solution. The solution was placed in the orbital shaker at 450 rpm to observe the colour change. In between the solution was taken the UV-visible Spectroscopy reading and it's was confirmed the synthesized nanoparticles. After 56 hours, this nanoparticle solution was centrifuged at 8000 rpm for 10 minutes. The pellet was collected for further research studies. Characterizations of Synthesized *Syzygium cumini*-Calcium Oxide Nanoparticles (CaO NPs). The progression of calcium oxide nanoparticle (CaO NP) formation was tracked at regular intervals using a UV-visible spectrophotometer, scanning across the wavelength range of 250-650 nm to confirm the appearance of characteristic absorption peaks associated with nano-scale CaO. Fourier Transform Infrared Spectroscopy (FTIR) was performed on the dried CaO NP sample to find the phytochemical groups involved in reducing and stabilizing the nanoparticles. X-ray Diffraction (XRD) XRD analysis was employed for the crystallographic investigation of the fabricated nanoparticle material, which verified various crystalline phases and lattice types of CaO nanoparticles. The particles were characterized for their external morphology, size, distribution and surface topography on scanning electron microscopy (SEM). Further EDX analysis was also performed to confirm the elemental composition of nanoparticles and incorporation of calcium and oxygen in the final material.

## Results and Discussion:

The antioxidant activity of *Syzygium cumini*-mediated calcium oxide nanoparticles (CaONPs) was done using the DPPH free radical scavenging assay. The nanoparticles showed a dose-dependent scavenging effect, with activity increasing from 41.36% at 10 μg/mL to 85.62% at 50 μg/mL. Being lower than control ascorbic acid (46.72-91.81%) the reducing power of the CaO NPs was significant given in Table 1. This strong DPPH scavenging activity was due to the high capping efficiency of the phytochemicals present in fruit extract, which allowed effective hydrogen or electron donation. Activity pattern is indicative for the synergistic antioxidant activity of both plant extracts and the high reactive surface area of nanoparticles. The CaONPs showed marked scavenging activity against hydrogen peroxide. Antioxidant activity increased from 47.13% (10 μg/mL) to 83.72% (50 μg/mL), which was close with the standard antioxidant (52.83% to 88.97%) as shown in Table 2. Hence, in vivo from the study, hydrogen peroxide is a ROS capable of generating hydroxyl radicals. The CaONPs demonstrated significant reducing activity, likely due to their metal-oxide surface facilitating electron transfer, neutralizing H_2_O_2_ before it decomposes into more harmful species. In the FRAP assay, the biosynthesized CaONPs exhibited a clear dose-dependent ferric-reducing activity, with absorbance values increasing from 52.69 to 80.32, closely comparable to the standard which ranged from 58.32 to 85.98 as depicted in Table 3. These findings demonstrate a strong reducing potential of the synthesized nanoparticles. FRAP assay measures the capacity to reduce Fe^3+^ to Fe^2+^, indicating the electron-donating ability of antioxidants. The substantial reducing power of CaONPs indicates that phenolic compounds on the nanoparticle surface play a crucial role in enhancing their redox properties. CaONPs demonstrated significant ABTS scavenging activity, increasing from 52.63% to 82.47%, closely comparable to ascorbic acid (57.65% to 87.61%) as shown in Figure 1 (see PDF). This assay reflects the antioxidant nature which includes both lipophilic and hydrophilic behavior. The excellent reducing effect demonstrates potent radical neutralizing ability of the *S. cumini* derived CaONPs. The *S. cumini* derived CaONPs. Possesses strong nitric oxide radical inhibition, from 54.75% to 81.96%, which is similar to the standard antioxidant (60.76% to 85.34%) as depicted in Figure 2 (see PDF). Nitric oxide, a reactive nitrogen species, plays a key role in chronic inflammation when produced in excess. The marked inhibitory effect demonstrated by CaONPs suggests their potential role in alleviating oxidative stress and inflammatory responses by limiting the formation of peroxynitrite and other harmful intermediates. This is similar to the findings observed in other fruit derived aqueous extract reported by Kumari *et al.* (2004) who found these extracts possess considerable antioxidant activity [6] This is also similar to study by Dathan *et al.* (2024) who conducted antioxidant activity of pomegranate peel extract [7]. Our study is also consistent with findings derived from research done by Kovoor *et al.* (2024) [8]. The antioxidant assays- hydrogen peroxide scavenging, DPPH, FRAP, ABTS, and nitric oxide inhibition together demonstrated that *Syzygium cumini*- mediated calcium oxide nanoparticles have strong and potent radical-scavenging and reducing abilities. This observation is consistent with earlier studies demonstrating that green-synthesized nanoparticles exhibit enhanced biological activity due to their phytochemical functional groups in the surface [9, 10]. With respect to all the assays, the nanoparticles showed a clear dose-dependent rise in activity, indicating that the antioxidant efficacy is strongly influenced by concentration of the nanomaterial The current study demonstrates that calcium oxide nanoparticles derived from *Syzygium cumini* fruit extract possess significant antioxidant activity, supporting the fact that phyto nanomaterials serve as effective bioactive agents. There have been several studies in the past which also confirmed that phytochemicals act as strong reducing and stabilizing agents, improving nanoparticle bioactivity and functional stability [8, 11]. Green syntheses of Nanoparticles have functional groups from the plant material used during synthesis. These phytochemical constituents play a dual role by stabilizing the nanoparticles and imparting redox-active constituents on their surface, which improves their capacity to donate electrons or hydrogen atoms for neutralization of reactive species [12]. In the ABTS assay and DPPH assay, the CaO NPs had potent free radical scavenging properties, which is comparable to the antioxidant activity of green-synthesized AgNPs derived from *Aegle marmelos, Cissus quadrangularis,* and *Coriandrum sativum* extracts [8, 10]. The FRAP assay also confirmed their reducing power which is observed by conversion of ferric to ferrous ions which is commonly seen with phenolic-rich natural antioxidants [7]. The capacity of CaO NPs to quench hydrogen peroxide and nitric oxide indicate their effectiveness against both reactive oxygen and reactive nitrogen species, which is similar with the antioxidant property seen in honey-derived functional nanocomposites and silver nanoparticle systems [9, 11]. The cooperation between the CaO nanoparticle core and the surrounding phytochemical layer likely contributes to improved stability, accelerated reaction kinetics, and amplified antioxidant potential. Such synergistic mechanisms are consistent with mechanism reported in numerous green-synthesized Nano systems in the literature [1, 11 and 12]. These properties position these nanoparticles promising candidates for biomedical applications - particularly in management of oxidative stress, development of wound healing formulations, and controlled drug delivery systems. This dose-dependent antioxidant activity seen in all assays points out that the nanoparticles can be modified to achieve a specific therapeutic strength. Moreover, the synthesis of such particles don't involve toxic chemicals, the resulting CaO NPs maintain a safety profile and biocompatible [5, 12]. The phytochemicals present in the *S. cumini* fruit extract predominantly polyphenols, flavonoids, and anthocyanins together play a significant role in stabilizing the nanoparticles not only reducing them but also contributing to their biological activity [8, 10]. The dimension of the CaO nanoscale particles also enhances surface interaction with free radicals, thereby improving their antioxidant profile. Collectively, the findings confirm that the green-synthesized CaO NPs preserve the inherent antioxidant properties of the fruit extract while enhanced reactivity attributable to Nan structuring a phenomenon comparable to the behavioural of carbon nanoparticles.

## Conclusion:

We show that CaONPs based *Syzygium cumini* fruit extract exhibited strong antioxidant activity across multiple assay systems, including notable free radical scavenging through hydrogen peroxide and nitric oxide inhibition, and significant considerable ferric-reducing power. Their broad-spectrum antioxidant spectrum, together with the eco-friendly and sustainability of green synthesis, supports their potential application in drug delivery systems, wound healing formulations, inflammation control, and therapies targeting oxidative stress. Future investigations focusing on in-vivo efficacy, toxicity profiling, and formulation stability will be essential to facilitate the clinical and translational advancement of these nanomaterials.

## Figures and Tables

**Table 1:** DPPH radical scavenging (%) Assay of CaONPs

**Concentration (μg/mL)**	**Standard**	**CaONPs**
10	46.72	41.36
20	58.65	53.68
30	71.85	67.35
40	82.64	77.91
50	91.81	85.62

**Table 2:** H_2_O_2_ scavenging (%) Activity

**Concentration (μg/mL)**	**Standard**	**CaONPs**
10	52.83	47.13
20	59.32	54.67
30	65.82	60.14
40	77.63	72.89
50	88.97	83.72

**Table 3:** FRAP values of CaONPs

**Concentration (μg/mL)**	**Standard**	**CaONPs**
10	58.32	52.69
20	70.44	67.34
30	76.82	71.56
40	81.52	77.92
50	85.98	80.32
